# Variability in Stroke Journeys: A Qualitative Study of Experiences of Persons With Stroke

**DOI:** 10.1111/hex.70902

**Published:** 2026-10-08

**Authors:** Asma Sabri, Heidi Dombestein, Peter Dieckmann, Stephen J. M. Sollid, Karina Aase

**Affiliations:** ^1^ Department of Quality and Health Technology, SHARE – Centre for Resilience in Healthcare, Faculty of Health Sciences University of Stavanger Stavanger Norway; ^2^ Department of Public Health, Faculty of Health Sciences University of Stavanger Stavanger Norway; ^3^ Department of Engineering Technology and Didactics, Section Leadership and Strategy Development Danish Technical University (DTU) Ballerup Denmark

**Keywords:** journey mapping, patient experiences, person‐centred, qualitative, stroke

## Abstract

**Background:**

Stroke recovery is highly individual and often involves multiple transitions across healthcare settings. While standardised stroke pathways aim to improve quality of care, less is known about how persons with stroke experience person‐centred practice throughout their stroke journeys.

**Aim:**

To explore how persons with stroke experience person‐centred practice throughout their stroke journeys.

**Methods:**

A qualitative study was conducted with persons who had experienced stroke in Norway. Data were collected through twelve semi‐structured interviews and one written response. Reflexive thematic analysis, narrative construction and patient journey mapping were used to explore their experiences across the first 3 months post‐stroke onset.

**Results:**

Participants' stroke journeys varied considerably in symptom presentation, needs and care experiences. Journey mapping visualised the differences in stroke journeys, healthcare interactions and experiences. Four themes were identified from the cross‐interview analysis: (1) Professional competence and relational care, (2) When the stroke pathway ends, the person's journey continues, (3) Standardised care versus individualised needs and (4) Participation across the journey. Experiences were generally positive in the hyperacute and acute phases and more variable following discharge, with participants describing limited follow‐up and access to rehabilitation, as well as unmet holistic needs.

**Conclusions:**

Persons with stroke experiences varied considerably throughout their stroke journeys, with discharge to home regarded as a stressful, insufficiently supported phase of care. Supporting continuity and individualised approaches to care should better meet the diverse needs of persons with stroke.

**Patient and Public Contribution:**

A user group consisting of persons with stroke and informal caregivers was recruited through a Norwegian stroke survivors association to be involved throughout the study. The user group consisted of seven persons, four females and three males, with ages ranging from their 40s to their 80s. The group included persons with stroke, persons with severe stroke, a person with aphasia, informal caregivers and the leader of the stroke association, who is also an informal caregiver. Two meetings, text communications and individual informal conversations took place with the user group to present and discuss the study design and obtain feedback on the interview content, format and procedures. The user group also supported participant recruitment. These participants took part in the interviews that form the basis of this study. Informal caregiver interviews were conducted in parallel as part of a related study. Preliminary findings were presented at the stroke survivors association's conference, and feedback was obtained.

## 1 Introduction

Stroke is one of the leading causes of death and disability worldwide [1] and places a substantial burden and costs on healthcare systems [2, 3]. Stroke is highly heterogeneous and has a lasting impact on people with stroke, often affecting multiple aspects of life, including physical, communicative, psychological, emotional and social aspects [4, 5], which results in a huge disruption of life [2]. For many persons with stroke, the journey from the onset of stroke to recovery is long and complex and includes multiple phases and settings. Advances in hyperacute treatment have improved survival and outcomes. However, ensuring high‐quality, person‐centred practices across stroke journeys remains a key challenge.

Stroke care is characterised by multiple transitions, and recovery is often described as dynamic and non‐linear rather than progressive [2], with people commonly experiencing fluctuations in physical, cognitive and emotional health. This individuality means that standardised approaches may be insufficient to meet the individual needs of persons with stroke, highlighting the importance of person‐centred practice, which takes into account the overall life situation, not just the immediate aspects related to the stroke itself. According to McCormack et al. [6], person‐centred practice emphasises building and fostering healthful relationships with service users and care providers. It is underpinned by values of personhood, self‐determination and sustained through cultures that support continuous practice development. In response to the burden of stroke, healthcare systems in multiple countries have developed structured clinical pathways and systems with the aim of improving the quality of stroke care, particularly in high‐income countries [7]. In Norway, as in many other countries, the stroke pathway follows the person from symptom onset through emergency contact and pre‐hospital care, acute diagnosis and treatment in hospital, admission to a dedicated stroke unit and on to rehabilitation and a 3‐month follow‐up in specialist healthcare [8]. The pathway covers the early stages of the stroke journey: the hyperacute and acute hospital phases, as well as the early subacute phase, usually including the transition back to community care. While the long‐term effects of stroke are well documented, this study focuses on the first 3 months post‐stroke onset, representing a dynamic phase of stroke care that offers high potential for recovery due to post‐stroke neuroplasticity [9]. This makes the early post‐stroke period critical for person‐centred stroke care.

Several studies and reviews have explored the experiences of persons with stroke and informal caregivers [2], yet limited research has explored persons with stroke's journeys across the stroke pathway, in which care is delivered across multiple and sometimes disconnected services. In a recent literature review exploring the global stroke context, we found gaps in continuity of care and communication in the early phases of recovery as ongoing challenges [10]. Quantitative studies evaluating the impact of standardised stroke pathways on functional outcomes and self‐perceived health in Norway have found that the pathway did not lead to improvements in functional outcomes, and that the attainment of care goals was not associated with self‐perceived health. However, the proportion of persons with stroke discharged directly home increased [11, 12]. While these studies provide important evidence regarding pathway outcomes, they offer limited insights into how persons with stroke experience the stroke pathway and the care provided throughout their stroke journeys.

Qualitative research on stroke in Norway has explored experiences from the perspectives of persons with stroke, informal caregivers and healthcare professionals using a range of methodological approaches, including interviews, focus groups and observational methods [13, 14, 15, 16]. One qualitative study identified unmet needs related to persons' insecurity during care transitions, lack of stroke rehabilitation expertise in primary care and ‘invisible’ stroke consequences, such as fatigue and cognitive impairment [17]. Although a body of qualitative research on stroke exists in Norway, the stroke pathway itself has primarily been evaluated using quantitative outcomes.

This study therefore aims to explore how persons with stroke experience person‐centred practice throughout their stroke journeys. The study is part of a larger project with the aim of developing guidance to support person‐centred practice during the first 3 months following stroke onset.

**Research Question:** *How do persons with stroke experience person‐centred practice throughout their stroke journeys during the first 3 months following stroke onset?*

## 2 Methodology

### 2.1 Design

This study followed a qualitative design [18] using semi‐structured interviews, narrative analysis and reflexive thematic analysis [19] to explore how persons with stroke experience person‐centred practice in their stroke journeys. The Consolidated Criteria for Reporting Qualitative Research (COREQ) were used to report the study [20]. A user group comprising persons with stroke and informal caregivers from a Norwegian stroke association was involved throughout the study and contributed to the study design, as well as to the interviews' content and format, to ensure their appropriateness for the target population. Patient and public involvement was reported in accordance with the GRIPP2 short‐form checklist [21].

### 2.2 Setting

In Norway, approximately 12,000 people are affected by stroke annually [8]. The Norwegian healthcare system is divided into two main levels of care: specialist health services managed by four regional health authorities and municipal health services managed by the municipalities. For persons with stroke, care is delivered across both levels throughout the stroke pathway. This study includes persons from two regional health authority regions.

For stroke care, the Norwegian Directorate of Health has developed a standardised stroke pathway, which includes two phases of care. The first phase of the stroke pathway was established in 2018 and includes the period from stroke onset until arrival at the stroke unit and any referral to stroke rehabilitation, spanning the first week after stroke onset, including the hyperacute and acute phases. In 2019, the pathway was expanded to include the entire 3 months post‐stroke period.

An important feature of the pathway is the emphasis on a thorough multidisciplinary assessment in the stroke unit, which forms the basis for decisions about each person's subsequent care pathway and whether it involves specialist inpatient rehabilitation, community rehabilitation, early supported discharge or follow‐up without rehabilitation. The pathway also recognises that standardised care must be balanced with individual needs and preferences [8].

### 2.3 Inclusion Criteria

Inclusion criteria were persons who had experienced a stroke within the last 3 years and were able to provide written consent and participate in a digital interview of approximately 1 h.

Persons with aphasia or severe disabilities were considered valuable to include in the study, and practical accommodations, such as the option to provide responses in written form or having a caregiver present during the interview, were offered to facilitate communication and participation.

### 2.4 Recruitment and Sample

Persons with stroke were recruited through the user group from a Norwegian stroke survivors association. An information letter describing the study was distributed through the association to recruit potential participants. In addition, a recruitment video was posted on the association's Facebook page, and information about the study was published in the association's member magazine. Individuals interested in the study were invited to provide their contact information. The study sought variation among participants with regard to age, stroke type, severity, gender and geographical area.

Thirteen persons who had experienced a stroke within the last 3 years were included in this study. The sample consisted of nine women and four men, ranging in age from 23 to 79 years. Twelve persons had an ischaemic stroke, and one person had a haemorrhagic stroke. Time since stroke ranged from 8 months to 2 years. All participants were from south‐eastern and western Norway.

### 2.5 Data Collection

Data were collected through individual semi‐structured interviews and in writing. Twelve participants took part in digital interviews, and one participant provided responses to the interview questions in a written format through a secure web‐based survey tool (Nettskjema, University of Oslo). Participants were given the option to have the interview through Microsoft Teams (Microsoft, 2025) or by phone call. In the case of phone calls, they were also contacted via Microsoft Teams for confidentiality purposes. The interviews were conducted between March and August 2025.

Interviews focused on capturing the participants' experiences of their stroke journeys during the first 3 months following stroke onset. The interview guide was developed based on the person‐centred practice framework [6] and covered topics such as shared decision‐making, engagement, healthcare professionals' skills and competences, and the care environment (Supplemental File 1).

Interviews were conducted by HD, with AS attending, and lasted approximately 1 h. Written consent forms were signed by participants prior to the interviews. Participants were also asked to consent verbally for the recordings at the beginning of the interviews. Participants were given enough time to express themselves and could ask for breaks when needed. Recordings were made using the Nettskjema Diktafon mobile application (University of Oslo, 2025), where audio was encrypted immediately upon recording.

### 2.6 Data Analysis

Recordings were transcribed using Whisper, an OpenAI transcription tool in Nettskjema, and were handled as confidential data throughout. Transcripts and connection keys were classified as confidential data and kept separately. For interviews with language issues, K.A. reviewed and corrected the transcripts. After anonymisation, transcripts were translated into English using a work account in Microsoft Co‐pilot approved for internal data.

Reflexive thematic analysis was first applied to each interview transcript. In parallel, narratives were constructed from each interview to facilitate a deeper understanding of participants' journeys and their timelines [22]. Thematic analysis and the narratives were both used to identify key themes specific to each participant (Table 2). A cross‐interview analysis was then conducted to identify common themes across participants' experiences [19] using NVivo 15. Patient journey mapping was conducted for three participants to provide a visual representation of their individual experiences [23]. The combination of these analytic approaches offered complementary and comprehensive insights into the data [24].

Inductive coding was performed by A.S. All authors checked and discussed the coding of three interviews during a joint meeting, and K.A. reviewed additional interviews throughout the analysis. Initial constructed narratives were also shared with co‐authors for feedback and to support familiarisation with the data.

### 2.7 Ethical Considerations

Written informed consent to participate in the study was obtained from participants prior to the interviews, and they were informed of their right to withdraw at any time. All data were anonymised and stored securely. This study was approved by the Norwegian Agency for Shared Services in Education and Research (SIKT), reference number 524469.

As persons with stroke may experience communication, cognitive and emotional challenges, particular attention was paid to their well‐being during recruitment and interviews. Interviews were adapted when needed, participants were given time to express themselves, and they were informed that they could pause or stop the interview at any time.

## 3 Results

### 3.1 Individual Experiences of the Stroke Journey

Table 1 presents an overview of the thirteen participants' experiences of their stroke journeys. The main finding across participants is the variability in symptoms, life circumstances and expectations of care. Therefore, what represented a gap in care differed between participants. For some, this was a diagnostic delay or lack of physical rehabilitation, whereas for others it was a lack of support for invisible impairments and challenges.

**Table 1:** Participants' individual experiences of their stroke journeys.

Person	Key themes
Person 1	– Delayed diagnosis associated with atypical symptoms – Limited emotional support and information – Challenges related to less visible consequences of stroke – Unreadiness for discharge
Person 2	– Rapid emergency assessment and treatment – Desire for early autonomy – Felt well taken care of – Good information flow and coordination
Person 3	– Not fitting into the stroke care system as a young person – Mental health concerns overlooked – Importance of informal caregivers' support – Variation in quality of care and healthcare professionals' attitudes
Person 4	– Positive experiences with home rehabilitation – Limited preparation for discharge and follow‐up – Need for conversation and information – Challenges related to home nursing services
Person 5	– Limited recollection of the early phases of care – Rehabilitation processes perceived as inflexible – Variation in healthcare professionals' competence and approaches
Person 6	– Critical failure in acute care – Concerns regarding stroke competence in some care encounters – Variable rehabilitation experiences – Financial consequences of stroke
Person 7	– Delayed diagnosis – Blood pressure measurement issues due to inadequate equipment – Challenges with home nursing services and inconsistent staff – Limited continuity and information
Person 8	– Delayed diagnosis and care – Limited involvement and information provision – Falls during hospitalisation – Rehabilitation perceived as delayed and insufficient
Person 9	– Rapid emergency response – Experience of isolation in the emergency room – Absence of follow‐up or rehabilitation – Ongoing unaddressed symptoms
Person 10	– Atypical stroke presentation nearly missed – Cognitive and sensory impact left without rehabilitation – Lack of post‐discharge support and loss of information – Self‐advocacy and peer support from a stroke association – Economic worries
Person 11	– Rapid physical recovery limiting access to care – Good acute care but limited support after discharge – Lack of psychological support – Importance of guidance on returning to work
Person 12	– Acute care perceived as positive – Absence of rehabilitation – Financial issues with having a stroke while on a minimum pension
Person 13	– 45‐minute ambulance delay; communication challenges related to aphasia – Shared room affecting sleep and rest – Unmet rehabilitation needs after discharge – Dealing with fatigue that was not addressed

### 3.2 Persons With Stroke's Journey Maps

To illustrate the variability of experiences across stroke journeys, journey maps were developed for three persons (Persons 1, 2 and 6), presented as principal actors in Figure 1. Each person is presented with a different colour throughout the map.

**Figure 1:**
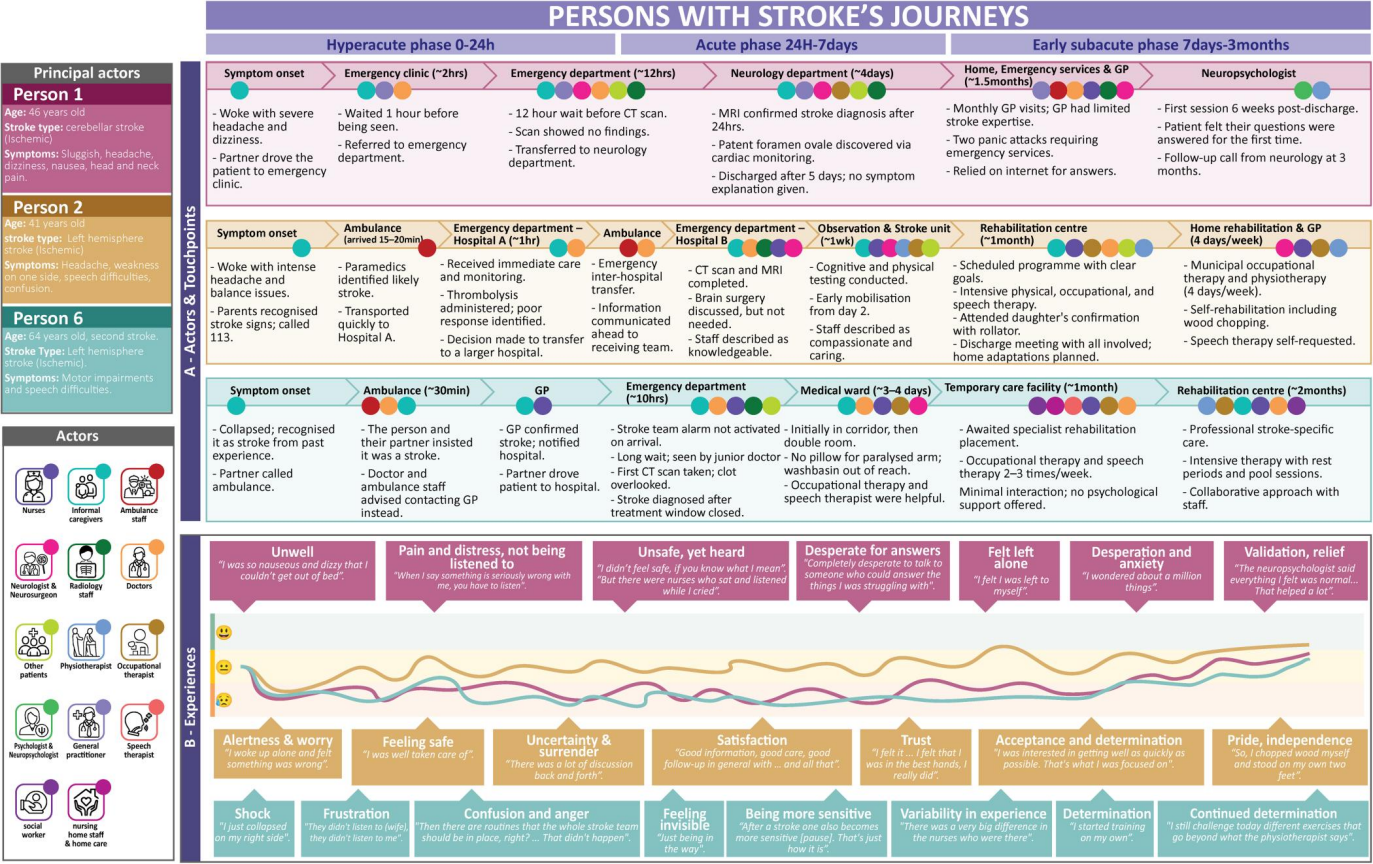
The patient journey as experienced by three persons with stroke during the first 3 months post‐stroke onset.

The journey maps are presented across the three phases of stroke recovery [9]: the hyperacute phase (0–24 h), the acute phase (24 h–7 days) and the early subacute phase (7 days–3 months). Section A of the map presents a horizontal timeline of care settings and services attended (touchpoints), starting from symptom onset (e.g., ambulance, emergency clinic) with an approximate duration noted for each phase. Under each care setting, main events are outlined, and different actors (healthcare professionals, informal caregivers) involved are presented with coloured dots. One dot may represent a single or multiple interactions. Section B summarises the emotional journey of each person as a continuous line according to their respective timeline, with key emotional states illustrated through quotes.

The sequence and duration of touchpoints, as well as the number and combination of actors, varied between the three persons, illustrating different levels of multidisciplinary involvement and coordination across their journeys. Emotional experiences also differed between the three persons, and at times they experienced prolonged periods of distress related to interactions with healthcare services.

Person 1, who had an atypical presentation of stroke and presented to the emergency clinic with headaches, dizziness and nausea, experienced less attention from healthcare professionals and significant delays. The person described having to manage significant consequences of stroke without appropriate information and support.

Person 2 had positive care experiences, which provided a valuable contrast for identifying what works well in stroke care. The person's journey illustrates the importance of rapid response, good communication, coordinated rehabilitation and effective community follow‐up.

Person 6 emphasised the importance of listening to the person and their informal caregiver and recognising their contribution to the diagnosis process. The person with stroke and their informal caregiver insisted that what they were experiencing was a stroke, as the person had previously had a stroke, but healthcare professionals decided that it was not urgent and asked them to contact their GP instead. The misdiagnosis resulted in significant impairments and frustration.

### 3.3 Common Experiences Across Stroke Journeys

In our cross‐interview analysis, we identified four themes, each capturing important elements of the persons with stroke's experiences of person‐centred practice throughout their stroke journeys (Table 2).

**Table 2:** Overview of themes and sub‐themes of persons with stroke's experiences of person‐centred practice throughout their stroke journeys.

1‐ Professional competence and relational care	The feeling of being taken seriously The importance of individual professionals
	Atypical presentation of stroke
2‐ When the stroke pathway ends, the person's journey continues	‘Left to myself’: The after‐discharge gap Access to rehabilitation and home care services
3‐ Standardised care versus individual needs	The ‘healthy’ stroke patient Unmet holistic needs
	Generic rather than individualised information provision
	The physical environment
4‐ Participation across the journey	Surrendering in the acute phase A more active role in the rehabilitation phase
	Important yet excluded: The role of informal caregivers

#### 3.3.1 Professional Competence and Relational Care

The hyperacute and acute phases of persons with stroke's journeys, which include the ambulance response and initial hospital admission (emergency department, stroke unit and neurology department), were generally experienced more positively compared to the later parts of their journeys. Persons with stroke described feeling safe and well cared for during this phase; however, others experienced challenges and delays.

##### 3.3.1.1 The Feeling of Being Taken Seriously

For persons with stroke, the speed and decisiveness of care in the acute phase were experienced as a form of care and were described positively. One person described arriving at the emergency department to find a five‐person stroke team waiting, which they found reassuring:

There were five people standing and receiving me … I experienced it very nicely. I was taken seriously, and I was taken care of. I wasn't scared or anything.

(Person 12)

Another person described the ambulance professionals in similar terms:

They understood that it was a stroke, so it was just about getting going. Very good. Really skilled.

(Person 2)

A person who lived alone on the tenth floor of an apartment building experienced that the safety alarm installed 1 month prior to the stroke became an important factor in their stroke journey:

I pressed the button, and then a person came here … An adult lady in the home care service. And just sat down here and looked at me … And called the ambulance right away. So, I was very soon off.

(Person 9)

There were also persons who described negative experiences during the early phases of care. Person 8 was sent home after their GP concluded that it had already been more than 4.5 h since the stroke had occurred:

The next day I fell and hurt myself so terribly that then we called the ambulance.

(Person 8)

##### 3.3.1.2 The Importance of Individual Professionals

Across all interviews, the experiences persons with stroke identified as most valuable were the individual relationships in which they were seen as whole persons by healthcare professionals. They described nurses who sat with them when they cried, stroked their hands, hugged them and asked whether they liked to read: ‘I couldn't praise them enough’ (Person 1).

They also described regular conversations with a night nurse about sports, grandchildren and other ordinary things, describing the nurse as the most trusted person during the hospital stay:

She was like between 50 and 60 years old. A bit like us. Had probably been a nurse for a long time … At night they have a bit more time than during the day. So, we sat [together], and I thought it was difficult that I couldn't talk, and she understood me very well. So that was reassuring for me. When she left in the morning, she stopped by me and said goodbye.

(Person 13)

Other Persons described similar experiences:

It was being seen at the hospital, actually. That's what… If I'm going to remember something after that, I think it is the kindness and the time and… I got… Everyone in a way had… They answered all the questions willingly.

(Person 11)

Persons with stroke also described the opposite experiences, including the feeling of being perceived as additional work, not getting a response after calling and being communicated with through forms requiring yes‐or‐no answers. They pointed to individual healthcare professionals as the reference point for good care and noted that the quality of care seemed to depend on who was on duty: ‘It was like a 50%–50% chance that it would be a good day’ (Person 5).

##### 3.3.1.3 Atypical Presentation of Stroke

Some persons with stroke experienced diagnostic delays or near‐missed diagnoses, and these persons had stroke presentations that did not conform with the classical FAST (Face, Arm, Speech, Time) symptoms. A person who had a cerebellar stroke presented with severe headache, nausea, vomiting and dizziness, and these symptoms did not receive sufficient attention from the emergency services. The person had to wait 24 h before undergoing an MRI, which confirmed the diagnosis, and noted that the stroke recognition poster displayed on the hospital wall depicted an older male presenting with FAST symptoms:

Maybe they should update their checkpoints. When it turns out it's not just older people who are affected by this. Or have the typical symptoms you read about on this poster.

(Person 1)

Another person had a stroke the morning following knee surgery, resulting in dizziness, blurred vision and ringing in the ears. According to the person, five of the six doctors who assessed them did not think they were having a stroke, as the symptoms were atypical:

Then about six doctors stood there and discussed what it was and wasn't. Five said it wasn't a stroke, and then there was one who said it is a stroke. Fortunately, it was him who said it was a stroke, who they listened to in the end.

(Person 10)

#### 3.3.2 When the Stroke Pathway Ends, the Person's Journey Continues

While persons with stroke reported a feeling of being taken seriously and a professional atmosphere in the acute phase, these experiences contrasted with what followed their hospital stay. Many described a gap after being discharged home and difficulties accessing rehabilitation.

##### 3.3.2.1 ‘Left to Myself’: The After‐Discharge Gap

Persons with stroke reported having to manage on their own after discharge despite ongoing disabilities and challenges. The transition from hospital to home was experienced as a sudden event with insufficient preparation and follow‐up:

When I was at the hospital, absolutely tip‐top…. But I have to say that when I left the hospital, I didn't have any check‐up. I was on my own.

(Person 11)

A person was informed of their discharge on a Friday afternoon, after 3 days in a stroke unit, which was too late for home care services to be arranged before the weekend. Discharge timing thus affected the continuity of care and coordination between hospital‐based care and community services.

The 3‐month follow‐up contact from the hospital occurred for most persons, either in person or by phone, sometimes delayed and was mostly described as brief and insufficient. One person was desperately waiting for the follow‐up to get answers about what to expect, but it was delayed for months despite them calling multiple times:

That conversation was important to me, and I was really waiting to get some clarity. I wanted to know where I was headed and what I could do.

(Person 4)

Other persons described the follow‐up as a brief phone call that did not answer their questions:

It was actually just… Is there any difference from when you left us, actually? And that was actually not anything more than that… Has it gotten any better? Has it gotten any worse? It wasn't so many minutes we talked.

(Person 10)

In contrast, another person had an extensive 3‐month follow‐up that involved multiple examinations and a conversation with a neurologist, and this experience was described positively.

##### 3.3.2.2 Access to Rehabilitation and Home Care Services

Training and rehabilitation were described as among the most important elements of persons with stroke's journeys: ‘Training. And it still is [the most important thing]’ (Person 8).

However, access to rehabilitation was often challenging. Some persons described referrals from the hospital and their GP to municipal rehabilitation services that did not result in any appointment for several months. In some cases, persons also reported receiving little or no rehabilitation during their hospital stay:

They got [a referral] from the hospital too that I should have a bit of rehabilitation… And that was also delivered to the rehabilitation centre, but it hasn't helped anything. I haven't gotten one appointment from that.

(Person 12)

I didn't get any training at the hospital. No. I tried to move myself, tried to walk, and then I fell.

(Person 8)

Some persons received rehabilitation but felt that it ended before they were ready. One person described how a home‐based rehabilitation programme was interrupted by healthcare professionals' holidays and ended while they still felt they needed support: ‘I didn't think it was finished, but I wasn't the one who decided’ (Person 7).

Persons with stroke who were able to access rehabilitation generally described positive experiences and perceived the quality of care as good. One person described the municipal rehabilitation team as supportive and available to answer questions. The person experienced person‐centred goal setting, as the team helped them get back to one of their favourite activities by including baking in the rehabilitation programme.

Some persons had home care because of their ongoing limitations following stroke. However, these services were insufficiently adapted to their needs and daily routines. These persons reported frequent changes in healthcare professionals, which they found tiring, making it difficult to build relationships and ask questions. Some persons described a home care service relying on written care plans and not responding to needs that were not specified in the assessment:

I was about to say… sorry to say it, but it's basically zero. They can't even take out the trash. There's basically nothing they can do. And I haven't felt that I could ask or talk to them about anything.

(Person 4)

#### 3.3.3 Standardised Care Versus Individual Needs

Persons with stroke described instances of mismatches between the standardised stroke care they received and their individual needs. Persons with invisible impairments reported that their challenges were not always recognised, that holistic needs beyond physical recovery were infrequently addressed, that information provision was often described as generic and that the physical environment was sometimes described as offering limited privacy and adaptation to individual circumstances.

##### 3.3.3.1 The ‘Healthy’ Stroke Patient

Some persons with stroke felt that their impairments were not taken seriously because they were not visibly disabled. Persons who could walk and talk after their stroke reported receiving less attention and follow‐up from healthcare services:

Absolutely not. And because I was so functionally fit, I was maybe too functional since I didn't have paralysis, and I could speak. So, I feel that the things I still struggle with today weren't… that they didn't end up in the right department, if you know what I mean.

(Person 1)

A person described this group as ‘healthy stroke patients’ and noted that they were left on their own after discharge. They also described others around them saying that there was nothing wrong with them and that they looked the same as before, while they were experiencing cognitive impairments such as short‐term memory problems and word‐finding difficulties:

But it's also a bit of the frustration, because… There's nothing wrong with you, [name]. Because you don't look any different or anything whatsoever. But I hear that it's quite common, from what I've understood.

(Person 10)

Another person spent a long time dealing with fine motor problems, fatigue and sensory changes without any professional support. In the absence of formal support for invisible impairments, persons described taking active roles in managing their recovery:

And it wasn't long before I went out into the snow with the big rollator and trained and trained.

(Person 9)

##### 3.3.3.2 Unmet Holistic Needs

Persons with stroke described a range of needs related to consequences that went beyond the physical consequences of their stroke, including cognitive, emotional and psychological consequences, changes to their relationships, as well as financial consequences. These experiences suggested that these needs were not always addressed within the stroke care pathway.

Psychological and emotional responses to stroke were described through experiences such as emotional lability, fear, grief and ongoing concern about the possibility of another stroke, consequences that were sometimes not named or addressed by healthcare professionals. Persons noted the value of having their experiences acknowledged and normalised:

But that's normal, he said. When you've had a stroke, you get very sad. But I think this is going to go very well with you, he said. So that meant a lot to me, then.

(Person 13)

Persons with stroke also reflected on the support that was offered to address emotional and psychological challenges. Some felt that the type or context of support did not align with their needs, such as being offered conversations with a hospital chaplain when they had hoped to speak with a psychologist, or receiving support in settings that limited privacy and openness.

Post‐stroke fatigue emerged as a challenging consequence of stroke. One person described it as ‘a monster with a thousand heads’, reflecting on how fatigue impacted many aspects of everyday life. Persons described limited information about post‐stroke fatigue during their interactions with healthcare professionals and reported discovering the condition through self‐directed reading and peer support.

Persons with stroke also wished that relationships, parenting and intimacy would be addressed by healthcare services:

But also, in relation to family and children and how to be a good parent.

(Person 2)

I would like to have a bit of sex with [Name], but I don't know how my muscles function in the lower body.

(Person 8)

For younger persons with stroke, unmet needs were also related to age and life circumstances:

I wanted, for example, to learn how I could do my hair with one hand, and no one could help me with that. They could, however, teach me how to change a tire.

(Person 3)

The person reflected that young persons with stroke are under‐researched and that information about welfare, work and education is lacking for people who are at the beginning of their adult lives.

##### 3.3.3.3 Generic Rather Than Individualised Information Provision

Persons with stroke identified several challenges related to the timing, content and delivery of information provision throughout their journeys. The way information was given sometimes did not match the person's capacity to receive it. They also described receiving information containing medical terminology they could not understand. As a result, some persons with stroke reported leaving the hospital with limited understanding of what had happened, what the consequences were or how to manage them:

I don't have more information than that it was a blood clot. That it went well. And now you have to take these medicines. I didn't know what a blood clot was.

(Person 9)

Communication challenges related to aphasia further influenced persons' experiences of information provision, which was described as brief, with little opportunity to ask questions or develop a fuller understanding.

One person received information that produced false optimism and was told that the brain would make new connections and get better, which was not the case. The person understood that this way of communication was an attempt to provide hope but expressed a preference for more honest and accurate information:

After… a year, when I was at the regular doctor again, then I said, can't you be so kind to call a spade a spade? What's happening now? Because that's how I see it. I see that I've been working for a year, and I actually see no progress. On the contrary, a couple of things have gone the opposite way.

(Person 10)

Experiences of discharge information varied, but the common situation was that persons left the hospital without fully understanding what had happened, what challenges they might encounter or what support was available. Written information was described as containing generic information not always relevant to individual persons. A person received a discharge brochure that contained information about walking frames even though the person did not have any physical impairments:

We got one of those folders to take home. But there it's also very… Aids from the assistive technology centre. With walking frames… Absolutely not [relevant].

(Person 1)

Persons described challenges related to information sharing between healthcare providers and the experience of having to repeat their history and circumstances to different healthcare professionals:

There's no transfer of information at all. Not any images, nothing…. It's not just between hospitals, but also internally at the hospital.

(Person 10)

##### 3.3.3.4 The Physical Environment

Persons with stroke described the physical environment throughout their journeys, where shared rooms were the norm in the hospital setting with limited space and privacy. Rehabilitation settings were described as being more spacious, with individual rooms as the norm as well as extensive training rooms and social areas offering more opportunities for socialising when desired.

Experiences of the hospital environment centred on the challenges associated with shared rooms, and persons described how the environment sometimes impacted their sleep, privacy and recovery. One person described lying beside a person who called out through the night for her deceased husband, while another person shared a room with a person who snored and rang the bell continuously, eventually requesting a solution from healthcare professionals. A person who had myalgic encephalomyelitis prior to the stroke described the experience of sensory overload in a shared room as exhausting. Similarly, another person described increased noise sensitivity after stroke:

I hobbled out of bed a couple of times to close the door after people who had been in with me … Some understood it, but others … they had worked at the hospital for 30 years, so they knew best.

(Person 10)

#### 3.3.4 Participation Across the Journey

Persons with stroke's experiences of involvement in decision‐making varied across different stages of their journeys. In the hyperacute phase, persons accepted and agreed with the clinical decision‐making on their behalf, with limited involvement. However, as their journeys progressed, their expectations of involvement increased. The involvement of informal caregivers was also a concern for persons as they were not always involved in care and decision‐making.

##### 3.3.4.1 Surrendering in the Acute Phase

Persons with stroke described the hyperacute phase as a period during which they had to trust the clinical decision‐making, with involvement limited to being informed due to their health condition:

So, I sort of surrender myself, so I felt that it went… everything went smoothly after that, that I…

(Person 5)

Some persons described moments when their capacity for participation was limited because of aphasia, shock or emotional overload. One person who was asked to consent to surgery while experiencing a stroke recalled:

It's quite intense when you're lying right in the middle of it and don't really understand what's happening, other than that this isn't good, so I also have to decide kind of whether… Whether I should operate or not. … I figure I was in good hands and was with professionals, so they made the right decisions along the way.

(Person 11)

##### 3.3.4.2 Variation in Participation in the Rehabilitation Phase

In rehabilitation settings, persons with stroke generally described more active involvement in decision‐making compared to the acute phase. Some recounted being invited to discuss their goals and priorities and felt that their preferences informed the rehabilitation plan:

They asked what my goals were, what was important for me to get started with again after the stroke. I mentioned baking.

(Person 4)

In contrast, other persons described limited involvement in goal setting. Some perceived the process as rigid and lacking dialogue, while others expressed uncertainty about how they could participate in their care:

I felt that there was little dialogue, little understanding for reasoning.

(Person 5)

##### 3.3.4.3 Important Yet Excluded: The Role of Informal Caregivers

Every person identified a family member or friend as highly important to their recovery. Informal caregivers provided emotional support, practical assistance, help with navigating the healthcare system and often played an important role during symptom onset and throughout the care journey. Persons reflected on how important such support was for their recovery:

They understood right away what it was. That's because [partner] works in healthcare and has knowledge about it. That's probably it.

(Person 5)

Had I not had my sister here, then I don't know what I should have done.

(Person 9)

One person noted the importance of their family and friends in their recovery and that individualised support and information access should be especially important ‘for those who don't have as resourceful relatives as others’.

Despite their important role, informal caregivers were not systematically included in the clinical encounters. A person's daughter had to wait in the corridor while the person was in the emergency department without receiving any information**:**

She was very anxious, and they couldn't find out how I was.

(Person 13)

One person described that their partner was not invited to the discharge meeting, while another person described how their partner had received a generic brochure for relatives of persons with stroke, with no direct engagement with healthcare professionals.

A person described how their informal caregivers had received an offer of conversational support during a specialist assessment conducted multiple months post‐stroke. The person reflected that such an offer was very important and should have come earlier:

I got an offer that my children wanted to talk to someone. Yes, because that … They too, the whole family. I get affected by this. It's a trauma for everyone.

(Person 11)

## 4 Discussion

The journeys described in this study present life‐changing events for the persons who participated and, together, illustrate the variability in how stroke affects them and their informal caregivers. This variability is less visible in the hyperacute phase but becomes more salient as the person moves from the hyperacute to the acute and subacute phases, creating a structural challenge at the transition between care settings: when the hospital's treatment ends, the person's journey continues and responsibility passes to healthcare professionals in different organisations, with different logics and priorities, each accountable for a large number of persons with their own needs. This generates—by structure—a tension between individual and organisational perspectives.

### 4.1 Variability in Care and Experiences

A noticeable result from our analysis was the variability in the experiences of persons with stroke regarding their stroke types, consequences and journeys. This variability was observed between and within different stroke services and reflects the complexity of stroke care with multiple transitions and interdependencies [25]. These findings are consistent with previous studies [26, 27], reflecting the heterogeneity of stroke as a condition.

Persons with stroke described their experiences during the hyperacute and acute phases as generally positive. These phases were characterised by speed, structure and professional competence emphasising the importance of specialised stroke units and timely interventions [28]. Our findings also suggest that what persons with stroke valued was not only clinical effectiveness but also the relational quality of care: being heard and seen beyond their stroke were among the moments they identified as most meaningful. This implies that the relational aspect of care should be treated as a core component of stroke care.

Rehabilitation was also described as being person‐centred by persons with stroke who had gained access to such services, as rehabilitation often provided more opportunity for individualised care after stabilisation of the acute condition. While this is a positive finding, Persons with stroke described difficulties in accessing such services. Current literature also documents difficulties in accessing follow‐up and rehabilitation services [27].

Taken together, the variation observed across the stroke journeys described in this study suggests that experiences of person‐centred practice are shaped by multiple factors. Relational person‐centred practice cannot rely on individual healthcare professionals' values and skills alone but requires organisational structures that support continuity of care, consistent practices and room for individualisation within standardised pathways [6].

### 4.2 When the Pathway Does Not Fit the Person

A pattern running through many of the sub‐themes in our analysis is that the stroke pathway as experienced by persons with stroke lacks adaptation to their needs and preferences. These findings point to the importance of considering how stroke services can better accommodate the diversity of persons with stroke [29].

Some persons experienced diagnostic delays or near‐missed diagnoses when their presentations did not match the FAST symptom profile, around which recognition protocols and public awareness campaigns have been organised. Posterior circulation strokes are known to have a substantially higher rate of misdiagnosis due to atypical symptoms such as dizziness, nausea and headaches [30, 31, 32]. This points to a need for updated recognition and diagnostic protocols that better reflect the diversity of stroke presentations, without compromising the simplicity that is often necessary in public awareness.

The ‘invisible’ persons with stroke, those who recover physically but retain significant cognitive, fatigue‐related, fine motor and emotional impairments, emerged as a group whose needs were sometimes unmet by the standardised stroke pathway. The experience of being discharged from the hospital with no formal support, despite cognitive fatigue and unprocessed trauma, is consistent with evidence from the literature documenting that cognitively and emotionally impaired persons with stroke without physical disability face particular barriers to accessing appropriate services [33, 34]. This gap matters as cognitive impairments affect a large majority of persons with stroke, but treatment options remain limited [35, 36]. Similarly, post‐stroke fatigue and depression are underdiagnosed and often not addressed by rehabilitation programmes [37], and unmet needs in this domain predict poorer quality of life [38].

Stroke is also becoming more prevalent in younger populations [39], and stroke services and rehabilitation programmes do not always match young persons' life circumstances and needs [40, 41]. For younger and working‐age persons in this study, information about welfare, studies and work was sometimes lacking, and rehabilitation programmes did not always meet their needs.

These findings show that while standardised stroke pathways offer consistency in care, they might be challenged by the diversity of persons with stroke. This points to the value of pathway models that are structured around the person's own priorities and goals, rather than task‐specific models [42]. Such person‐centred interventions based on self‐determination may help address the gap identified in this study, especially for persons whose needs fall outside the standardised pathways.

### 4.3 The Need for Person‐Centred Care Transitions

The transition from hospital to home was experienced by persons as a sudden discontinuity in care and was described as stressful and sometimes forced. Persons experienced a sudden expectation to take full responsibility for their situation after having had a passive role during their hospital stay. Similar findings have been documented across multiple studies [27, 43]. Longitudinal research suggests that the transition is not a single event but a process with different phases, where new challenges emerge over time [44]. Recent research has identified insufficient information provision, fragmented services and inequitable access to rehabilitation as features of the post‐discharge period for persons with stroke [45].

Information at discharge was sometimes experienced as generic by the participants in this study. A meta‐ethnographic study on persons with stroke's unmet needs after discharge found that lack of information availability and suitability was one of the two main issues for stroke persons alongside the inadequacy of care and services [46]. There is a need for solutions to optimise information provision for persons with stroke, considering the heterogeneity and the multimorbidity within this population [47]. Standardised discharge information is unlikely to meet the varied needs of persons with stroke.

Taken together, these findings suggest that a person‐centred transition from hospital to home would require preparing persons and their informal caregivers for discharge, and providing information that is tailored to individual needs, readiness and capacity, and implementing systems that support information transfer might be beneficial in these circumstances [48].

Some limitations to this study need to be considered. Although the group of persons with stroke included in this study provided a good range of age, gender and stroke types, thirteen participants do not necessarily cover the range of experiences of persons with stroke. The time since stroke ranged from 8 months to 2 years, which might have affected participants' recollection of the first 3 months following stroke onset. The study includes only one person with haemorrhagic stroke, and, given that ischaemic stroke and haemorrhagic stroke can differ significantly in their presentation and recovery, the findings primarily reflect the experiences of persons with ischaemic stroke.

## 5 Conclusion

This study found considerable variation in persons' experiences of their stroke journeys, both between and within healthcare services. Experiences were most consistently positive in the acute phase and became more varied as their journeys extended beyond the hospital. Person‐centred practices depended mostly on individual healthcare professionals rather than organisational structures, and the transition from hospital to home was experienced as a withdrawal of care despite ongoing needs.

The standardised stroke pathway as experienced by persons with stroke addressed a narrow definition of the person with stroke. This definition has left younger persons, persons with atypical presentations of stroke, and those with invisible impairments at risk of having their needs unmet. Underlying these findings is a structural challenge: responsibility for the person's journey passes between institutions with different logics and priorities, each accountable for a large number of persons with stroke. Meeting the diversity of persons with stroke's needs will therefore require organisational structures that support continuity and individualisation, and not only the efforts of individual healthcare professionals.

## Author Contributions

**Asma Sabri:** conceptualization, investigation, writing – original draft, methodology, validation, visualization, writing – review and editing, project administration, formal analysis, supervision, data curation, resources. **Heidi Dombestein:** investigation, validation, writing – review and editing, resources. **Peter Dieckmann:** validation, writing – review and editing, supervision. **Stephen J. M. Sollid:** validation, writing – review and editing, supervision. **Karina Aase:** validation, methodology, conceptualization, formal analysis, writing – review and editing, supervision, data curation, resources, project administration, funding acquisition.

## Ethics Statement

This study was approved by the Norwegian Agency for Shared Services in Education and Research (SIKT), ref. number: 524469.

## Consent

Written informed consent was obtained from all participants prior to participation in the study.

## Conflicts of Interest

The authors declare no conflicts of interest.

## Declaration of AI Use

The translation of transcripts after anonymisation and the language editing for this manuscript were performed using Microsoft Co‐pilot with University of Stavanger access approved for internal data.

## Supporting information


Supporting File

## Data Availability

Due to privacy concerns, full de‐identified transcripts cannot be shared. However, summaries of the analyses and the study codebook are available upon request.
