# Supplementary material for: Variability in Stroke Journeys: A Qualitative Study of Experiences of Persons With Stroke
Source: Health Expect. 2026 Oct 8;29(5):e70902. doi: 10.1111/hex.70902 (PMC13648015; doi:10.1111/hex.70902)
Supplement: Supplementary file 1 [file HEX-29-e70902-s001.docx]

**Additional file 1: Interview guide**

| **Background information** | Age  Occupation/Education  Marital status  Time of stroke |
| --- | --- |
| **Care environment** | **At which different healthcare settings did you receive treatment and care?**  **Can you describe these different settings (environments)?**   - How did you experience the physical environment? - Did the physical environment meet your needs?   - For example, privacy? Being able to move around as you wished? - What was it like for you in the different settings?   - The atmosphere on the ward? Busy or unhurried?   - Did you interact with many or few staff members? How did you experience that?   - How did you feel you were cared for when transferred between departments or levels of care? |
| **Person-Centred Processes** | **How did you experience the treatment and care you received throughout your journey?**   - Beyond your physical health, did the care you received take into account other aspects of your health (emotional, social, and spiritual)? - Were your beliefs, values, and preferences taken into consideration in the care you received? - How actively involved were you in your own care? - What would have helped you feel more engaged in your care? - How did your needs change over the first three months (pre-hospital care, stroke unit, rehabilitation)? |
| **Prerequisites** **(Healthcare Professionals****’ Attributes)** | **What are your thoughts about the healthcare professionals who cared for you?**   - How did you perceive the healthcare professionals’ competence, skills, and values?   - Were there any skills or areas of expertise that you felt were lacking? - Did you get a sense of how healthcare professionals collaborated in your care? - Can you describe an example of when a healthcare professional showed compassion and presence? What about when they seemed less present or genuine?   - Did healthcare professionals seem interested in what you had to say? - Person-centred practice emphasises the importance of shared power, where the person is an equal partner and should feel empowered.   - What are your thoughts on this? Is this something you experienced, or not? |
| **Involvement and Shared Decision-Making** | **How would you describe your involvement in your care journey during the first three months after your stroke?**   - How were you involved in decision-making?   - Did you receive the information you needed to make informed choices?   - Were there formal meetings, or was information provided more informally/ad hoc?   - Were you involved in decisions regarding medical treatment, procedures, assessments, or goal setting (for example, rehabilitation goals)?   - How much influence did you have over your daily activities and routines? - Were your family members/caregivers involved? If so, how? - Did you feel included in decisions about your care, and were your preferences taken into account? - What would you consider to be the critical elements for involvement in each phase?   (1) Acute phase: pre-hospital care and stroke unit  (2) Follow-up phase: rehabilitation and community/home care |
| **Overall Experience** | **What stands out most to you when you think about your entire experience throughout your care journey?**   - What was most important to you? - Is there anything we have not asked about that you feel is important for us to know? |
